# Comparison of Sodium-Glucose Cotransporter-2 Inhibitor and Dipeptidyl Peptidase-4 Inhibitor on the Risks of New-Onset Atrial Fibrillation, Stroke and Mortality in Diabetic Patients: A Propensity Score-Matched Study in Hong Kong

**DOI:** 10.1007/s10557-022-07319-x

**Published:** 2022-02-10

**Authors:** Sharen Lee, Jiandong Zhou, Keith Sai Kit Leung, Abraham Ka Chung Wai, Kamalan Jeevaratnam, Emma King, Tong Liu, Wing Tak Wong, Carlin Chang, Ian Chi Kei Wong, Bernard Man Yung Cheung, Gary Tse, Qingpeng Zhang

**Affiliations:** 1 Diabetes Research Unit, Cardiovascular Analytics Group, Hong Kong, China-UK Collaboration China; 2 Nuffield Department of Medicine, University of Oxford, Oxford, UK; 3 Emergency Medicine Unit, Faculty of Medicine, The University of Hong Kong, Hong Kong, China; 4 Faculty of Health and Medical Sciences, University of Surrey, Guildford, UK; 5 Tianjin Key Laboratory of Ionic-Molecular Function of Cardiovascular Disease, Department of Cardiology, Tianjin Institute of Cardiology, Second Hospital of Tianjin Medical University, Tianjin, 300211 China; 6 School of Life Sciences, State Key Laboratory of Agrobiotechnology (CUHK), The Chinese University of Hong Kong, Hong Kong, China; 7 Division of Neurology, Department of Medicine, Queen Mary Hospital, Hong Kong, China; 8 Department of Pharmacology and Pharmacy, University of Hong Kong, Hong Kong, China; 9 Division of Clinical Pharmacology and Therapeutics, Department of Medicine, The University of Hong Kong, Hong Kong, China; 10 Kent and Medway Medical School, Canterbury, UK; 11 School of Data Science, City University of Hong Kong, Hong Kong, China

**Keywords:** Sodium-glucose cotransporter-2 inhibitors, Dipeptidyl peptidase-4 inhibitors, Diabetes, Atrial fibrillation, Stroke, Cardiovascular mortality, All-cause mortality

## Abstract

**Objective:**

To compare the effects of sodium-glucose cotransporter 2 inhibitors (SGLT2Is) and dipeptidyl peptidase-4 inhibitors (DPP4Is) on adverse outcomes in diabetic patients in Hong Kong.

**Methods:**

This was a retrospective population-based cohort study of type 2 diabetes mellitus patients (***n*** = 72**,**746) treated with SGLT2I or DPP4I between January 1, 2015, and December 31, 2020, in Hong Kong. Patients with exposure to both DPP4I and SGLT2I therapy, without complete demographics or mortality data, or who had prior atrial fibrillation (AF) were excluded. The study outcomes were new-onset AF, stroke/transient ischemic attack, cardiovascular mortality and all-cause mortality. Propensity score matching (1:1 ratio) between SGLT2I and DPP4I users was performed.

**Results:**

The unmatched study cohort included ﻿21,713 SGLT2I users and 39,510 DPP4I users (total: *n* = 61,233 patients; 55.37% males, median age: 62.7 years [interquartile range (IQR): 54.6–71.9 years]). Over a median follow-up of 2030 (IQR: 1912–2117) days, 2496 patients (incidence rate [IR]: 4.07%) developed new-onset AF, 2179 patients (IR: 3.55%) developed stroke/transient ischemic attack, 1963 (IR: 3.20%) died from cardiovascular causes and 6607 patients (IR: 10.79%) suffered from all-cause mortality. After propensity score matching (SGLT2I: *n* = 21,713; DPP4I: *n* = 21,713), SGLT2I users showed lower incidence of new-onset AF (1.96% vs. 2.78%, standardized mean difference [SMD] = 0.05), stroke (1.80% vs. 3.52%, SMD = 0.11), cardiovascular mortality (0.47% vs. 1.56%, SMD = 0.11) and all-cause mortality (2.59% vs. 7.47%, SMD = 0.22) compared to DPP4I users. Cox regression found that SGLT2I users showed lower risk of new-onset AF (hazard ratio [HR]: 0.68, 95% confidence interval [CI]: [0.56, 0.83], *P* = 0.0001), stroke (HR: 0.64, 95% CI: [0.53, 0.79], *P* < 0.0001), cardiovascular mortality (HR: 0.39, 95% CI: [0.27, 0.56], *P* < 0.0001) and all-cause mortality (HR: 0.44, 95% CI: [0.37, 0.51], *P* < 0.0001) after adjusting for significant demographics, past comorbidities, medications and laboratory tests.

**Conclusions:**

Based on real-world data of type 2 diabetic patients in Hong Kong, SGLT2I use was associated with lower risk of incident AF, stroke/transient ischemic attack, and cardiovascular and all-cause mortality outcomes compared to DPP4I use.

**Supplementary Information:**

The online version contains supplementary material available at 10.1007/s10557-022-07319-x.

## Introduction

Type 2 diabetes mellitus is an increasingly prevalent metabolic disease, with significant cerebrovascular and cardiovascular complications, including stroke, heart failure (HF) and myocardial infarction [1]. Diabetic patients are associated with a 2.5-fold increased risk of ischemic stroke, and the risk is dependent on glycemic levels [2, 3]. Atrial fibrillation (AF) is a known risk factor for ischemic stroke, and diabetes mellitus increases the risk of AF through a combination of structural and electrical cardiac remodeling [4–7].

The potential protective effects of novel antidiabetic agents on cardiovascular and cerebrovascular diseases are increasingly becoming the focus of research [8–11]. Recent studies have shown the association between the use of sodium-glucose cotransporter-2 inhibitors (SGLT2Is) and lower risks of all-cause and cardiovascular mortality amongst diabetic patients in comparison to other antidiabetic agents [12–14]. Similarly, dipeptidyl peptidase-4 inhibitor (DPP4I) use has been reported to reduce the risk of major cardiovascular adverse events, including stroke, though its effect on heart failure remains controversial [15–17]. However, few studies have compared SGLT2Is and DPP4Is in their effects on stroke and AF. A recent multinational study reported lower stroke risk amongst SGLT2I users compared to DPP4I users in a group of patients with type 2 diabetes mellitus [13]. However, Hong Kong was not included in its Asia-Pacific data analysis, and the risk of AF has not been examined. Therefore, to elucidate the cerebrovascular effects of SGLT2I and DPP4I, the present study aims to evaluate the risk of ischemic stroke, AF, cardiovascular and all-cause mortality between SGLT2I and DPP4I users in the Hong Kong population.

## Methods

### Study Design and Population

This study obtained ethics approval from the Institutional Review Board of the University of Hong Kong/Hospital Authority Hong Kong West Cluster and The Joint Chinese University of Hong Kong–New Territories East Cluster Clinical Research Ethics Committee. This was a retrospective, territory-wide cohort study of type 2 diabetes mellitus patients with SGLT2I/DPP4I use between January 1, 2015, and December 31, 2020, in Hong Kong. Patients with any SGLT2I/DPP4I use during the aforementioned period were enrolled and followed up until December 31, 2020, or until death. Patients with both DPP4I and SGLT2I use, with less than 1 month of SGLT2I or DPP4I exposure or with prior AF diagnosis were excluded. The patients were identified from the Clinical Data Analysis and Reporting System (CDARS), a citywide database that centralizes patient information from individual local hospitals to establish comprehensive medical data, including clinical characteristics, disease diagnosis, laboratory results and drug treatment details. The system has been previously used by both our team and other teams in Hong Kong on epidemiological research [18, 19], including those on diabetes [20].

Clinical and biochemical data were extracted for the present study. Patients’ demographics include gender and age of initial SGLT2I/DPP4I use. Prior comorbidities were extracted based on the International Classification of Diseases Ninth Revision (ICD-9) codes (Supplementary Table 1). The Charlson comorbidity index was also calculated. Mortality was recorded using the International Classification of Diseases Tenth Revision (ICD-10) coding. ICD-10 codes I00–I09, I11, I13 and I20–I51 were used to identify cardiovascular mortality outcomes. Medication histories were also extracted, including the use of metformin, sulfonylurea, insulin, acarbose, thiazolidinedione, glucagon-like peptide-1 receptor agonists, glimepiride, glibenclamide, gliclazide, glipizide, anticoagulants, statins and fibrates. Baseline laboratory data, including complete blood count, biochemical tests, glycemic and lipid profiles were extracted.

### Outcomes and Statistical Analysis

The study outcomes were new-onset ischemic stroke/transient ischemic attack, new-onset AF, all-cause and cardiovascular mortality. Mortality data were obtained from the Hong Kong Death Registry, a population-based official government registry with the registered death records of all Hong Kong citizens linked to CDARS. Descriptive statistics are used to summarize baseline clinical and biochemical characteristics of patients with SGLT2I and DPP4I use. For baseline clinical characteristics, the continuous variables were presented as median (95% confidence interval [CI]/interquartile range [IQR]) or mean (standard deviation [SD]) and the categorical variables were presented as total number (percentage). Continuous variables were compared using the two-tailed Mann-Whitney *U* test, whilst the two-tailed Chi-square test with Yates’ correction was used to test 2 × 2 contingency data.

Propensity score matching with 1:1 ratio between SGLT2I and DPP4I users based on demographics, CHA2DS2-VASc score, Charlson comorbidity index, prior comorbidities, use of different medication classes (including other antidiabetic drugs), and baseline hemoglobin A1c (HbA1c) and fasting glucose tests were performed using the nearest neighbor search strategy with the caliper as 0.1. For the missing baseline covariates upon admission, multiple imputation by chained equations (MICE) was employed in the treatment and control groups. Each missing value of laboratory data was imputed 20 times using other variables that might have an impact on the study outcomes. Univariable and multivariable Cox regression models were used to identify significant risk predictors for the study outcomes. Competing risk analysis models (cause-specific and sub-distribution) were considered. A standardized mean difference (SMD) of less than 0.2 between the treatment groups post-weighting was considered an adequate balance. The hazard ratio (HR), 95% CI and *P* value were reported. Cumulative incidence curves were used to illustrate the difference in the time-to-adverse event in the SGLT2I and DPP4I groups visually. Statistical significance is defined as a *P* value <0.05. Statistical analyses were performed with RStudio software (version: 1.1.456) and Python (version: 3.6). Stata software (version 13.0) was used for propensity score matching.

## Results

### Basic Characteristics of the Study Cohort

**Fig. 1:**
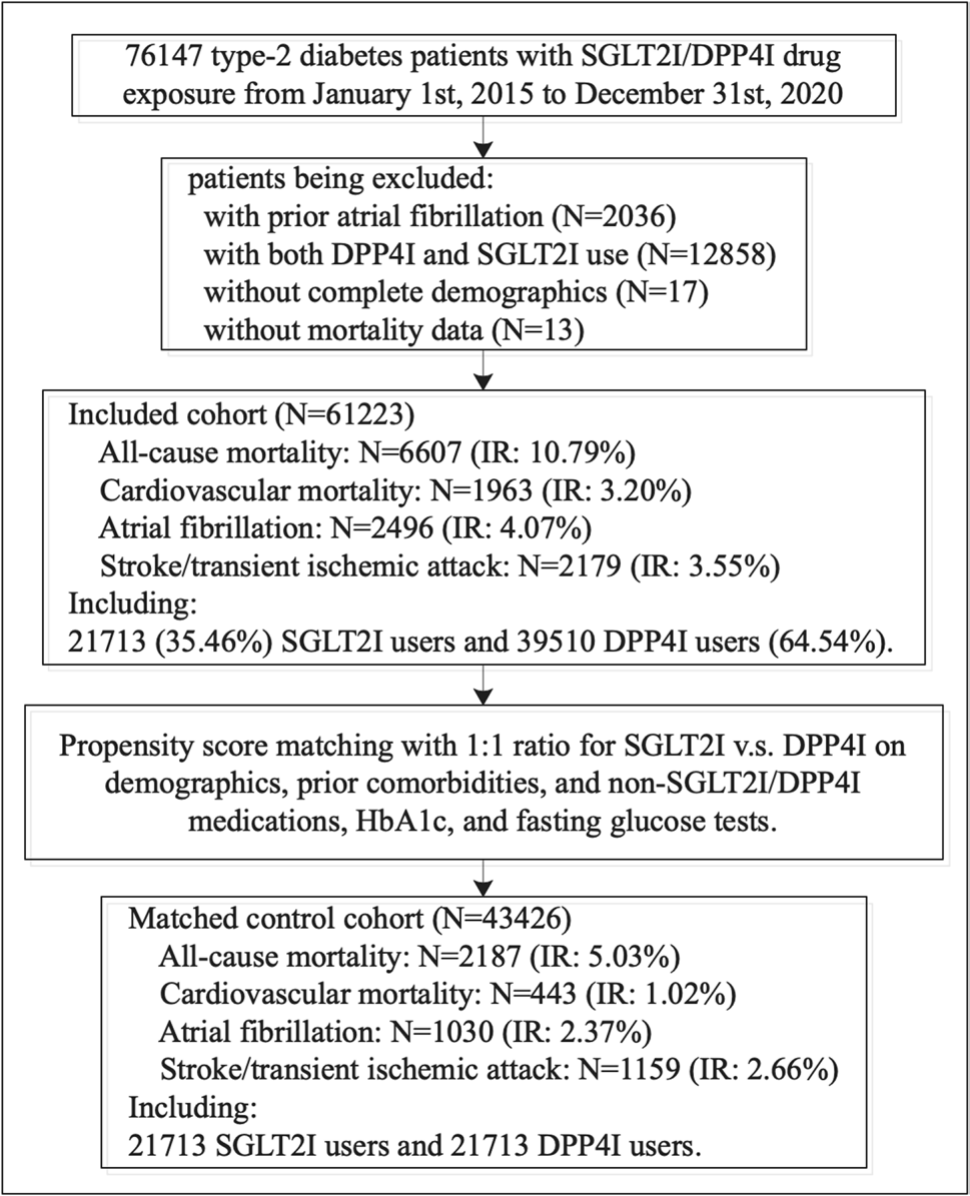
Procedures for data processing for the study cohort

**Fig. 2:**
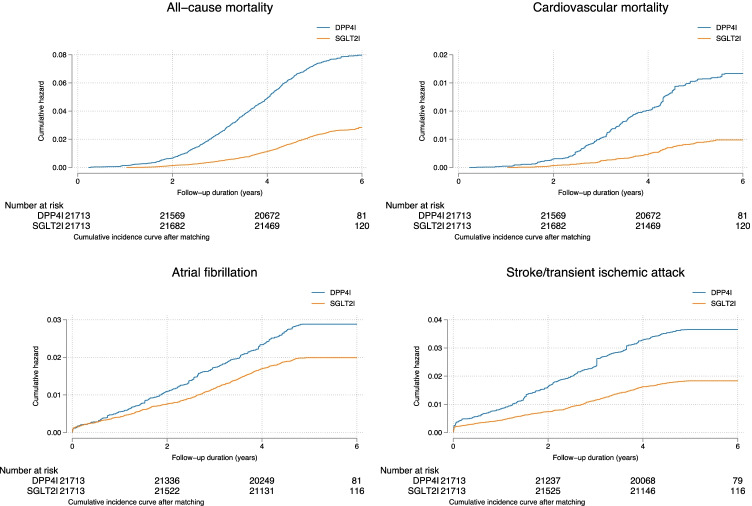
Cumulative incidence curves for new-onset atrial fibrillation, stroke/transient ischemic attack, cardiovascular mortality and all-cause mortality stratified by SGLT2I or DPP4I use in the matched cohort

This study cohort included 61,233 patients with type 2 diabetes mellitus (55.37% males, median age: 62.7 years [interquartile range (IQR): 54.56–71.92 years]). Of these, ﻿21,713 were SGLT2I users (35.46%) and 39,510 were DPP4I users (64.54%). The breakdown of the number of patients on individual SGLT2Is was as follows: 12,824 (29.53%) on dapagliflozin, 4600 (10.59%) on empagliflozin, 4994 (11.50%) on canagliflozin and 2510 (5.77%) on ertugliflozin. Over a median follow-up duration of 2030 (IQR: 1912–2117) days, 2496 patients (IR: 4.07%) developed new-onset AF, 2179 patients (IR: 3.55%) developed stroke/transient ischemic attack, and 6607 patients (IR: 10.79%) died from any cause, among which 1963 (IR: 3.20%) died from cardiovascular diseases (Fig. 1). The cumulative incidence of the adverse outcomes compared between SGLT2I and DPP4I users after propensity score matching is summarized by cumulative incidence curves in Fig. 2.

The baseline demographics and clinical characteristics of DPP4I and SGLT2I users before and after 1:1 propensity score matching are shown in Table 1. The medications and laboratory tests at baseline are detailed in Supplementary Table 2. The distributions of density as a function of the propensity score before and after matching using a caliper of 0.1 are shown in Supplementary Fig. 1. Both before and after matching, SGLT2I users showed lower incidence of new-onset AF (before: 1.96% vs. 5.23%, SMD = 0.18; after: 1.96% vs. 2.78%, SMD = 0.05), stroke (before: 1.80% vs. 4.52%, SMD = 0.16; after: 1.80% vs. 3.52%, SMD = 0.11), all-cause mortality (before: 2.59% vs. 15.29%, SMD = 0.46; after: 2.59% vs. 7.47%, SMD = 0.22) and cardiovascular mortality (before: 0.47% vs. 4.70%, SMD = 0.27; after: 0.47% vs. 1.56%, SMD = 0.11) compared to DPP4I users.

**Table 1:** Baseline and clinical characteristics of patients with SGLT2I or DPP4I use before and after propensity score matching

Characteristics	Before matching			SMD	After matching			SMD
	All (*N* = 61,223) mean (SD); *N* or count (%)	SGLT2I users (*N* = 21,713) mean (SD); *N* or count (%)	DPP4I users (*N* = 39,510) mean (SD); *N* or count (%)		All (*N* = 43,426) mean(SD); *N* or count (%)	SGLT2I users (*N* = 21,713) mean (SD); *N* or count (%)	DPP4I users (*N* = 21,713) mean (SD); *N* or count (%)	
Demographics								
Male gender	33,900 (55.37%)	13,011 (59.92%)	20,889 (52.87%)	0.14	26,006 (59.88%)	13,011 (59.92%)	12,995 (59.84%)	0
Female gender	27,323 (44.62%)	8702 (40.07%)	18,621 (47.12%)	0.14	17,420 (40.11%)	8702 (40.07%)	8718 (40.15%)	0
Baseline age, years	63.0 (12.9); *n* = 61,223	57.6 (11.3); *n* = 21,713	66.0 (12.7); *n* = 39,510	0.7*	58.4 (11.3); *n* = 43,426	57.6 (11.3); *n* = 21,713	59.1 (11.2); *n* = 21,713	0.13
<50	8693 (14.19%)	4789 (22.05%)	3904 (9.88%)	0.34*	8831 (20.33%)	4789 (22.05%)	4042 (18.61%)	0.09
[50–60]	16,895 (27.59%)	7803 (35.93%)	9092 (23.01%)	0.29*	15,463 (35.60%)	7803 (35.93%)	7660 (35.27%)	0.01
[60–70]	17,816 (29.10%)	6425 (29.59%)	11,391 (28.83%)	0.02	13,121 (30.21%)	6425 (29.59%)	6696 (30.83%)	0.03
[70–80]	11,120 (18.16%)	2219 (10.21%)	8901 (22.52%)	0.34*	4838 (11.14%)	2219 (10.21%)	2619 (12.06%)	0.06
>80	6705 (10.95%)	480 (2.21%)	6225 (15.75%)	0.49*	1177 (2.71%)	480 (2.21%)	697 (3.21%)	0.06
Past comorbidities								
Charlson standard comorbidity index	2.1 (1.5); *n* = 61,223	1.5 (1.2); *n* = 21,713	2.4 (1.6); *n* = 39,510	0.61*	1.6 (1.3); *n* = 43,426	1.5 (1.2); *n* = 21,713	1.6 (1.3); *n* = 21,713	0.08
Diabetes with chronic complication	696 (1.13%)	246 (1.13%)	450 (1.13%)	0	488 (1.12%)	246 (1.13%)	242 (1.11%)	0
Diabetes without chronic complication	1100 (1.79%)	464 (2.13%)	636 (1.60%)	0.04	888 (2.04%)	464 (2.13%)	424 (1.95%)	0.01
Gastrointestinal bleeding	1435 (2.34%)	394 (1.81%)	1041 (2.63%)	0.06	774 (1.78%)	394 (1.81%)	380 (1.75%)	0
Gout	1674 (2.73%)	469 (2.15%)	1205 (3.04%)	0.06	918 (2.11%)	469 (2.15%)	449 (2.06%)	0.01
Heart failure	1525 (2.49%)	405 (1.86%)	1120 (2.83%)	0.06	797 (1.83%)	405 (1.86%)	392 (1.80%)	0
Hyperlipidemia	1670 (2.72%)	783 (3.60%)	887 (2.24%)	0.08	1493 (3.43%)	783 (3.60%)	710 (3.26%)	0.02
Hypertension	14,330 (23.40%)	5043 (23.22%)	9287 (23.50%)	0.01	9948 (22.90%)	5043 (23.22%)	4905 (22.59%)	0.02
Hypoglycemia	495 (0.80%)	52 (0.23%)	443 (1.12%)	0.11	104 (0.23%)	52 (0.23%)	52 (0.23%)	0
Ischemic heart disease	5867 (9.58%)	2744 (12.63%)	3123 (7.90%)	0.16	5081 (11.70%)	2744 (12.63%)	2337 (10.76%)	0.06
Liver diseases	1329 (2.17%)	659 (3.03%)	670 (1.69%)	0.09	1250 (2.87%)	659 (3.03%)	591 (2.72%)	0.02
Acute myocardial infarction	1596 (2.60%)	715 (3.29%)	881 (2.22%)	0.06	1405 (3.23%)	715 (3.29%)	690 (3.17%)	0.01
Peripheral vascular disease	460 (0.75%)	119 (0.54%)	341 (0.86%)	0.04	238 (0.54%)	119 (0.54%)	119 (0.54%)	0
Renal diseases	1152 (1.88%)	118 (0.54%)	1034 (2.61%)	0.17	236 (0.54%)	118 (0.54%)	118 (0.54%)	0
VT/VF/SCD	132 (0.21%)	66 (0.30%)	66 (0.16%)	0.03	132 (0.30%)	66 (0.30%)	66 (0.30%)	0
Anemia	2523 (4.12%)	495 (2.27%)	2028 (5.13%)	0.15	977 (2.24%)	495 (2.27%)	482 (2.21%)	0
Overweight	426 (0.69%)	345 (1.58%)	81 (0.20%)	0.15	658 (1.51%)	345 (1.58%)	313 (1.44%)	0.01
Cancer	1676 (2.73%)	442 (2.03%)	1234 (3.12%)	0.07	874 (2.01%)	442 (2.03%)	432 (1.98%)	0
Stroke/transient ischemic attack	1857 (3.03%)	517 (2.38%)	1340 (3.39%)	0.06	1011 (2.32%)	517 (2.38%)	494 (2.27%)	0.01
Outcomes								
All-cause mortality	6607 (10.79%)	564 (2.59%)	6043 (15.29%)	0.46*	2187 (5.03%)	564 (2.59%)	1623 (7.47%)	0.22*
Cardiovascular mortality	1963 (3.20%)	103 (0.47%)	1860 (4.70%)	0.27*	443 (1.02%)	103 (0.47%)	340 (1.56%)	0.11
Atrial fibrillation	2496 (4.07%)	426 (1.96%)	2070 (5.23%)	0.18	1030 (2.37%)	426 (1.96%)	604 (2.78%)	0.05
Stroke/transient ischemic attack	2179 (3.55%)	393 (1.80%)	1786 (4.52%)	0.16	1159 (2.66%)	393 (1.80%)	766 (3.52%)	0.11

*for SMD ≥ 0.2, SD: standard deviation, SCD: sudden cardiac death, VF: ventricular fibrillation, VT: ventricular tachycardia, SGLT2I: sodium-glucose cotransporter-2 inhibitor, DPP4I: dipeptidyl peptidase-4 inhibitor, ACEI: angiotensin-converting enzyme inhibitors, ARB: angiotensin-receptor blockers, CV: coefficient of variation

### Cox Regression, Competing Risk and Sensitivity Analyses

The detailed results of univariable Cox regression in the unmatched and matched cohorts are shown in Supplementary Table 3 (AF and stroke/transient ischemic attack) and Supplementary Table 4 (cardiovascular and all-cause mortality). Compared to DPP4I users, SGLT2I users showed lower risks of new-onset AF (HR: 0.68, 95% CI: [0.56, 0.83], *P* = 0.0001), stroke (HR: 0.64, 95% CI: [0.53, 0.79], *P* < 0.0001), all-cause mortality (HR: 0.44, 95% CI: [0.37, 0.51], *P* < 0.0001) and cardiovascular mortality (HR: 0.39, 95% CI: [0.27, 0.56], *P* < 0.0001) after adjusting for significant demographics, past comorbidities, medications and biomarkers (Table 2). These findings were confirmed by competing risk analyses using cause-specific and sub-distribution hazard models (Table 3). Sensitivity analyses using a one-year lag time (Supplementary Table 5) and approaches based on the propensity score (Supplementary Table 6) were performed. All of these analyses demonstrated the association between SGLT2I use and lower risks of incident AF, stroke/transient ischemic attack, cardiovascular mortality, and all-cause mortality compared to DPP4I use.

**Table 2:** Multivariable Cox regression to identify significant predictors of atrial fibrillation, stroke/transient ischemic attack, cardiovascular mortality and all-cause mortality in the matched cohort

	Atrial fibrillation HR [95% CI]; *P* value	Stroke transient ischemic attack HR [95% CI]; *P* value	Cardiovascular mortality HR [95% CI]; *P* value	All-cause mortality HR [95% CI]; *P* value
Model 1	0.76 [0.68–0.87]; <0.0001***	0.52 [0.46–0.59]; <0.0001***	0.34 [0.27–0.42]; <0.0001***	0.37 [0.33–0.40]; <0.0001***
Model 2	0.76 [0.67–0.86]; <0.0001***	0.51 [0.45–0.58]; <0.0001***	0.34 [0.27–0.42]; <0.0001***	0.36 [0.33–0.40]; <0.0001***
Model 3	0.83 [0.73–0.94];0.0031**	0.54 [0.47–0.61]; <0.0001***	0.40 [0.32–0.51]; <0.0001***	0.40 [0.36–0.44]; <0.0001***

* for *P* ≤ 0.05, ** for *P* ≤ 0.01, *** for *P* ≤ 0.001; HR: hazard ratio; CI: confidence interval; SGLT2I: sodium-glucose cotransporter-2 inhibitor; DPP4I: dipeptidyl peptidase-4 inhibitor; IR: incidence rate

Model 1 adjusted for significant demographics

Model 2 adjusted for significant demographics and past comorbidities

Model 3 adjusted for significant demographics, past comorbidities and non-SGLT2I/DPP4I medications

**Table 3:** Competing risk analyses in the matched cohort

Models	Outcomes	SGLT2I vs. DPP4I HR [95% CI];P value
Cause-specific hazard models		
	Atrial fibrillation	0.90 [0.76–1.00]; 0.0451*
	Stroke/transient ischemic attack	0.61 [0.52–0.72]; <0.0001***
	Cardiovascular mortality	0.57 [0.40–0.81]; 0.0015**
	All-cause mortality	0.33 [0.21–0.66]; 0.0140*
Sub-distribution hazard models		
	Atrial fibrillation	0.90 [0.76–1.00]; 0.0151*
	Stroke/transient ischemic attack	0.61 [0.52–0.72]; <0.0001***
	Cardiovascular mortality	0.29 [0.19–0.43]; <0.0001***
	All-cause mortality	0.37 [0.31–0.43]; <0.0001***

* for *P* ≤ 0.05, ** for *P* ≤ 0.01, *** for *P* ≤ 0.001; SGLT2I: sodium-glucose cotransporter-2 inhibitors; DPP4I: dipeptidyl peptidase-4 inhibitors; HR: hazard ratio; CI: confidence interval

The number of patients on individual SGLT2Is on study outcomes in the matched cohort is summarized in Table 4. Due to changes in medication switching, some patients were exposed to more than one SGLT2I. Exposure to both empagliflozin and canagliflozin (*n* = 1596) was the most common, followed by canagliflozin and ertugliflozin (*n* = 646), dapagliflozin and empagliflozin (*n* = 575), dapagliflozin and canagliflozin (*n* = 252), dapagliflozin and ertugliflozin (*n* = 140), and dapagliflozin, canagliflozin and ertugliflozin (*n* = 3).

**Table 4:** Descriptive statistics and event rates for patients on different individual SGLT2I agents

SGLT2I drugs	All SGLT2I users (*N* = 21,713) Mean (SD); *N* or count(%)	New-onset atrial fibrillation (*N* = 1030)	Stroke/transient ischemic attack (*N* = 1159)	Cardiovascular mortality (*N* = 443)	All-cause mortality (*N* = 2187)
		Mean (SD); *N* or count(%)	Mean (SD); *N* or count(%)	Mean (SD); *N* or count(%)	Mean (SD); *N* or count(%)
Dapagliflozin	12,824 (29.53%)	228 (22.13%)	219 (18.89%)	68 (15.34%)	352 (16.09%)
Empagliflozin	4600 (10.59%)	101 (9.80%)	88 (7.59%)	15 (3.38%)	99 (4.52%)
Canagliflozin	4994 (11.50%)	102 (9.90%)	79 (6.81%)	17 (3.83%)	119 (5.44%)
Ertugliflozin	2510 (5.77%)	54 (5.24%)	54 (4.65%)	13 (2.93%)	66 (3.01%)

* for P ≤ 0.05, ** for *P* ≤ 0.01, *** for *P* ≤ 0.001; SD: standard deviation, HR: hazard ratio, CI: confidence interval, SGLT2I: sodium-glucose cotransporter-2 inhibitors, DPP4I: dipeptidyl peptidase-4 inhibitors

## Discussion

The major finding of the present study is that amongst type 2 diabetes patients, SGLT2I users have a lower risk for new-onset AF, stroke, cardiovascular mortality and all-cause mortality compared with DPP4I users after propensity score matching on Cox regression. These findings were confirmed by competing risk analysis.

To our knowledge, this is the first study with a head-to-head comparison of new-onset stroke and AF risk of SGLT2I and DPP4I users with type 2 diabetes mellitus. Whilst the superior effects of SGLT2I against DPP4I in reducing all-cause and cardiovascular mortality have been reported, few studies have compared the effects of the two drug classes in their effects on stroke and AF specifically [12]. However, the meta-analysis had a smaller sample size, and confounders were not adjusted for. Recently, a multinational observational study on diabetic patients involving 13 countries demonstrated significantly lower all-cause mortality and stroke risk in SGLT2I users in comparison to DPP4I users, which is supportive of the present findings [13]. A network meta-analysis of randomized controlled trials found that DPP4Is did not reduce major adverse cardiovascular events (MACE) or mortality compared to placebo and were associated with higher risks of MACE, HF-related hospitalizations and all-cause mortality compared to SGLT2Is [21].

By contrast, the effect of SGLT2Is on AF is more controversial. The risk of AF was numerically lower in all and statistically lower in some clinical trials comparing diabetic SGLT2I users and controls [22–25]. A recent meta-analysis of 16 randomized controlled trials demonstrated a significant reduction of AF and atrial flutter amongst diabetic SGLT2I and placebo users [11]. Although the CVD-REAL Nordic study on cardiovascular mortality and morbidity in patients with type 2 diabetes following initiation of SGLT2I therapy versus other glucose-lowering drugs demonstrated a nonsignificant difference in stroke and AF between dapagliflozin and diabetic DPP4I users, their analysis did not adjust for clinical or biochemical confounders, or a history of the two diseases, respectively [26]. Animal studies have also demonstrated that SGLT2Is can prevent atrial remodeling, which is an important pathogenic mechanism of AF [27, 28]. Thus, the findings from the present study, which is based on more stringent patient selection and confounder adjustments and is better supported by the large-scale trials on SGLT2I, are likely a better reflection of the clinical circumstances. In patients, the protective effects of SGLT2Is are likely mediated through weight loss, diuretic effect, decreased blood pressure, and better glucose and lipid profile [29]. It should be noted that it remains unclear whether the cardiovascular outcomes are similar across individual SGLT2Is, particularly when large-scale randomized controlled trials comparing different SGLT2Is against placebo use yielded different results, which is also demonstrated in the present study when individual SGLT2Is were analyzed.

Existing reports on the cardiovascular effects of DPP4I remain controversial. Nonsignificant differences in cardiovascular mortality, AMI and ischemic stroke between diabetic DPP4I users and controls were reported by both the Cardiovascular Outcomes Study of Alogliptin in Patients With Type 2 Diabetes and Acute Coronary Syndrome (EXAMINE) and the Saxagliptin Assessment of Vascular Outcomes Recorded in Patients With Diabetes Mellitus–Thrombolysis in Myocardial Infarction 53 (SAVOR-TIMI53) trials [30, 31]. Hospitalization for acute HF increased in the SAVOR-TIMI53 trial, though the results were not replicated by other trials [30–32]. A nonsignificant elevation in the risk of HF-related hospitalization was reported in a meta-analysis summarizing these clinical trials, with substantial heterogeneity across trials using different DPP4Is, suggesting that unique features of specific DPP4I may have individual cardiovascular effects [33].

Nevertheless, the differences in outcomes between DPP4I and SGLT2I identified in this study are likely attributable to their adverse and protective effects on the cardiovascular system, respectively [34, 35]. However, there appears to be a gap between the protective actions of DPP4I on cardiac remodeling and their overall benefit, or lack thereof, clinically for patients, and these discrepancies should be examined in the future [36, 37]. The greater protective effects of SGLT2I against AF and stroke suggest that patients at risk for the two conditions should be prescribed an SGLT2I instead of a DPP4I as a part of the individualized management plan for patients with type 2 diabetes mellitus.

### Limitations

Several limitations should be noted for the present study. First of all, given its observational nature, there is inherent information bias due to under-coding, coding errors and missing data. Also, patients’ drug compliance can only be assessed indirectly through prescription refills, which are ultimately not a direct measurement of drug exposure. Secondly, residual and post-baseline confounding may be present despite robust propensity-matching, particularly with the unavailability of information on lifestyle cardiovascular risk factors. Patients’ drug exposure duration has not been controlled, which may affect their risk against the study outcomes. Moreover, data on blood glucose and HbA1c were largely missing, and thus the glycemic control in the patients cannot be adequately assessed. Additionally, the occurrence of AF out of the hospital is not accounted for, though non-sustained AF that does not result in hospital admission can be considered subclinical.

## Conclusions

Based on real-world data on type 2 diabetic patients in Hong Kong, SGLT2I use was associated with lower risks of incident AF, stroke, and cardiovascular and all-cause mortality outcomes compared to DPP4I use.

## Supplementary Information

ESM 1 (DOCX 68 kb)

## Data Availability

Deidentified data are available from the corresponding authors.
