# Supplementary material for: Comparison of Sodium-Glucose Cotransporter-2 Inhibitor and Dipeptidyl Peptidase-4 Inhibitor on the Risks of New-Onset Atrial Fibrillation, Stroke and Mortality in Diabetic Patients: A Propensity Score-Matched Study in Hong Kong
Source: Cardiovasc Drugs Ther. 2022 Feb 10;37(3):561–9. doi: 10.1007/s10557-022-07319-x (PMC10164005; doi:10.1007/s10557-022-07319-x)
Supplement: Supplementary file 1 — (DOCX 68 kb) [file 10557_2022_7319_MOESM1_ESM.docx]

**Supplementary Figure 1. Propensity score matching comparisons for SGLT2I v.s. DPP4I before and after 1:1 matching using the nearest neighbor search strategy with a caliper of 0.1.**

**Supplementary Table 1. ICD-9 codes for comorbidities and ICD-10 codes for outcomes.**

| Diabetes mellitus 250 250.01 250.02 250.03 250.1 250.11 250.12 250.13 250.2 250.21 250.22 250.23 250.3 250.31 250.32 250.33 250.4 250.41 250.42 250.43 250.5 250.51 250.52 250.53 250.6 250.61 250.62 250.63 250.7 250.71 250.72 250.73 250.8 250.81 250.82 250.83 250.9 250.91 250.92 250.93 |
| --- |
| Renal diseases 582 582 582.1 582.2 582.4 582.8 582.81 582.89 582.9 583 583 583.1 583.2 583.4 583.6 583.7 585 585.1 585.2 585.3 585.4 585.5 585.6 585.9 586 588 588 588.1 588.8 588.81 588.89 588.9 |
| Acute myocardial infarction 410 410.01 410.02 410.1 410.11 410.12 410.2 410.21 410.22 410.3 410.31 410.32 410.4 410.41 410.42 410.5 410.51 410.52 410.6 410.61 410.62 410.7 410.71 410.72 410.8 410.81 410.82 410.9 410.91 410.92 |
| Hypertension 401 401.1 401.9 402 402.01 402.1 402.11 402.9 402.91 403 403.01 403.1 403.11 403.9 403.91 404 404.01 404.02 404.03 404.1 404.11 404.12 404.13 404.9 404.91 404.92 404.93 405 405.01 405.09 405.1 405.11 405.19 405.9 405.91 405.99 437.2 |
| Heart failure 428 428 428.1 428.2 428.2 428.21 428.22 428.23 428.3 428.3 428.31 428.32 428.33 428.4 428.4 428.41 428.42 428.43 428.9 398.91 402.01 402.11 402.91 404.01 404.03 404.11 404.13 404.91 404.93 |
| Atrial fibrillation 427.31 429.4 |
| Liver diseases 456 456.1 456.2 572.2 572.3 572.4 572.8 571.4 571.5 571.6 |
| Peripheral vascular disease 250.7 443.9 443 443.1 443.2 443.21 443.22 443.23 443.24 443.29 443.8 443.81 443.82 443.89 441 443.9 785.4 V43.4 |
| Stroke/transient ischemic attack 435 435.1 435.2 435.3 435.8 435.9 433.81 433.91 434 436 437 437.1 433.31 433.01 434.01 434.1 434.11 434.9 434.91 437.2 437.3 437.4 437.5 437.6 437.7 437.8 437.9 430 431 432 432.1 432.9 |
| Gastrointestinal bleeding 531 531.2 531.4 531.6 532 532.2 532.4 532.6 533 533.2 533.4 533.6 534 534.2 534.4 534.6 535.01 535.11 535.21 535.31 535.41 535.51 535.61 535.71 562.02 562.03 562.12 562.13 569.3 569.85 569.86 578 578.1 578.9 |
| Ischemic heart disease 410.01 410.02 410.1 410.11 410.12 410.2 410.21 410.22 410.3 410.31 410.32 410.4 410.41 410.42 410.5 410.51 410.52 410.6 410.61 410.62 410.7 410.71 410.72 410.8 410.81 410.82 410.9 410.91 410.92 411 411.1 411.8 411.81 411.89 413 413.1 413.9 414 414.01 414.02 414.03 414.04 414.05 414.06 414.07 414.1 414.11 414.12 414.19 414.2 414.3 414.4 414.8 414.9 410 412 |
| Cancer 140 140.1 140.3 140.4 140.5 140.6 140.8 140.9 141 141.1 141.2 141.3 141.4 141.5 141.6 141.8 141.9 142 142.1 142.2 142.8 142.9 143 143.1 143.8 143.9 144 144.1 144.8 144.9 145 145.1 145.2 145.3 145.4 145.5 145.6 145.8 145.9 146 146.1 146.2 146.3 146.4 146.5 146.6 146.7 146.8 146.9 147 147.1 147.2 147.3 147.8 147.9 148 148.1 148.2 148.3 148.8 148.9 149 149.1 149.8 149.9 150 150.1 150.2 150.3 150.4 150.5 150.8 150.9 151 151.1 151.2 151.3 151.4 151.5 151.6 151.8 151.9 152 152.1 152.2 152.3 152.8 152.9 153 153.1 153.2 153.3 153.4 153.5 153.6 153.7 153.8 153.9 154 154.1 154.2 154.3 154.8 155 155.1 155.2 156 156.1 156.2 156.8 156.9 157 157.1 157.2 157.3 157.4 157.8 157.9 158 158.8 158.9 159 159.1 159.8 159.9 160 160.1 160.2 160.3 160.4 160.5 160.8 160.9 161 161.1 161.2 161.3 161.8 161.9 162 162.2 162.3 162.4 162.5 162.8 162.9 163 163.1 163.8 163.9 164 164.1 164.2 164.3 164.8 164.9 165 165.8 165.9 170 170.1 170.2 170.3 170.4 170.5 170.6 170.7 170.8 170.9 171 171.2 171.3 171.4 171.5 171.6 171.7 171.8 171.9 172 172.1 172.2 172.3 172.4 172.5 172.6 172.7 172.8 172.9 173 173.01 173.02 173.09 173.1 173.11 173.12 173.19 173.2 173.21 173.22 173.29 173.3 173.31 173.32 173.39 173.4 173.41 173.42 173.49 173.5 173.51 173.52 173.59 173.6 173.61 173.62 173.69 173.7 173.71 173.72 173.79 173.8 173.81 173.82 173.89 173.9 173.91 173.92 173.99 174 174.1 174.2 174.3 174.4 174.5 174.6 174.8 174.9 175 175.9 176 176.1 176.2 176.3 176.4 176.5 176.8 176.9 179 180 180.1 180.8 180.9 181 182 182.1 182.8 183 183.2 183.3 183.4 183.5 183.8 183.9 184 184.1 184.2 184.3 184.4 184.8 184.9 185 186 186.9 187 187.1 187.2 187.3 187.4 187.5 187.6 187.7 187.8 187.9 188 188.1 188.2 188.3 188.4 188.5 188.6 188.7 188.8 188.9 189 189.1 189.2 189.3 189.4 189.8 189.9 190 190.1 190.2 190.3 190.4 190.5 190.6 190.7 190.8 190.9 191 191.1 191.2 191.3 191.4 191.5 191.6 191.7 191.8 191.9 192 192.1 192.2 192.3 192.8 192.9 193 194 194.1 194.3 194.4 194.5 194.6 194.8 194.9 195 195.1 195.2 195.3 195.4 195.5 195.8 200 200.01 200.02 200.03 200.04 200.05 200.06 200.07 200.08 200.1 200.11 200.12 200.13 200.14 200.15 200.16 200.17 200.18 200.2 200.21 200.22 200.23 200.24 200.25 200.26 200.27 200.28 200.3 200.31 200.32 200.33 200.34 200.35 200.36 200.37 200.38 200.4 200.41 200.42 200.43 200.44 200.45 200.46 200.47 200.48 200.5 200.51 200.52 200.53 200.54 200.55 200.56 200.57 200.58 200.6 200.61 200.62 200.63 200.64 200.65 200.66 200.67 200.68 200.7 200.71 200.72 200.73 200.74 200.75 200.76 200.77 200.78 200.8 200.81 200.82 200.83 200.84 200.85 200.86 200.87 200.88 201 201.01 201.02 201.03 201.04 201.05 201.06 201.07 201.08 201.1 201.11 201.12 201.13 201.14 201.15 201.16 201.17 201.18 201.2 201.21 201.22 201.23 201.24 201.25 201.26 201.27 201.28 201.4 201.41 201.42 201.43 201.44 201.45 201.46 201.47 201.48 201.5 201.51 201.52 201.53 201.54 201.55 201.56 201.57 201.58 201.6 201.61 201.62 201.63 201.64 201.65 201.66 201.67 201.68 201.7 201.71 201.72 201.73 201.74 201.75 201.76 201.77 201.78 201.9 201.91 201.92 201.93 201.94 201.95 201.96 201.97 201.98 202 202.01 202.02 202.03 202.04 202.05 202.06 202.07 202.08 202.1 202.11 202.12 202.13 202.14 202.15 202.16 202.17 202.18 202.2 202.21 202.22 202.23 202.24 202.25 202.26 202.27 202.28 202.3 202.31 202.32 202.33 202.34 202.35 202.36 202.37 202.38 202.4 202.41 202.42 202.43 202.44 202.45 202.46 202.47 202.48 202.5 202.51 202.52 202.53 202.54 202.55 202.56 202.57 202.58 202.6 202.61 202.62 202.63 202.64 202.65 202.66 202.67 202.68 202.7 202.71 202.72 202.73 202.74 202.75 202.76 202.77 202.78 202.8 202.81 202.82 202.83 202.84 202.85 202.86 202.87 202.88 202.9 202.91 202.92 202.93 202.94 202.95 202.96 202.97 202.98 203 203.01 203.02 203.1 203.11 203.12 203.8 203.81 203.82 204 204.01 204.02 204.1 204.11 204.12 204.2 204.21 204.22 204.8 204.81 204.82 204.9 204.91 204.92 205 205.01 205.02 205.1 205.11 205.12 205.2 205.21 205.22 205.3 205.31 205.32 205.8 205.81 205.82 205.9 205.91 205.92 206 206.01 206.02 206.1 206.11 206.12 206.2 206.21 206.22 206.8 206.81 206.82 206.9 206.91 206.92 207 207.01 207.02 207.1 207.11 207.12 207.2 207.21 207.22 207.8 207.81 207.82 208 208.01 208.02 208.1 208.11 208.12 208.2 208.21 208.22 208.8 208.81 208.82 208.9 208.91 208.92 196 196.1 196.2 196.3 196.5 196.6 196.8 196.9 197 197.1 197.2 197.3 197.4 197.5 197.6 197.7 197.8 198 198.1 198.2 198.3 198.4 198.5 198.6 198.7 198.8 198.81 198.82 198.89 199 199.1 |
| Obesity 278.01 278 278 |
| Hypertension 401 401.1 401.9 402 402.01 402.1 402.11 402.9 402.91 403 403.01 403.1 403.11 403.9 403.91 404 404.01 404.02 404.03 404.1 404.11 404.12 404.13 404.9 404.91 404.92 404.93 405 405.01 405.09 405.1 405.11 405.19 405.9 405.91 405.99 437.2 |
| Hypoglycemia 251.2 |
| Hypotension 458 458 458.1 458.2 458.21 458.29 458.8 458.9 |
| Anaemia 280 280.1 280.8 280.9 281 281.1 281.2 281.3 281.4 281.8 281.9 282.2 282.3 282.8 282.9 283 283.1 283.11 283.19 283.2 283.9 284 284.01 284.09 284.1 284.11 284.12 284.19 284.81 284.9 285 285.1 285.2 285.21 285.22 285.29 285.3 285.8 285.9 |
| Overweight 278 278 278 278.01 278.02 278.03 278.1 278.2 278.3 278.4 278.8 |
| Gout 274 274.01 274.02 274.03 274.1 274.11 274.19 274.8 274.81 274.82 274.89 274.9 |
| Ventricular tachycardia/ventricular fibrillation/sudden cardiac death 410 410.01 410.02 410.1 410.11 410.12 410.2 410.21 410.22 410.3 410.31 410.32 410.4 410.41 410.42 410.5 410.51 410.52 410.6 410.61 410.62 410.7 410.71 410.72 410.8 410.81 410.82 410.9 410.91 410.92 427.01 427.1 427.4 427.4 427.41 427.42 427.5 427.5 427.69 798 798.1 798.2 |

**Supplementary Table 2. Baseline medications before and after propensity score matching.**

* for SMD$\geq$0.2; SD: standard deviation; SCD: sudden cardiac death; VF: ventricular fibrillation; VT: ventricular tachycardia; SGLT2I: sodium glucose cotransporter-2 inhibitor; DPP4I: dipeptidyl peptidase-4 inhibitor; ACEI: angiotensin converting enzyme inhibitors; ARB: angiotensin-receptor blockers; CV: coefficient of variation.

| **Characteristics** | **Before matching** |  |  | **SMD** | **After matching** |  |  | **SMD** |
| --- | --- | --- | --- | --- | --- | --- | --- | --- |
|  | **All (N=61223) Mean(SD);N or Count(%)** | **SGLT2I users (N=21713) Mean(SD);N or Count(%)** | **DPP4I users (N=39510) Mean(SD);N or Count(%)** |  | **All (N=43426) Mean(SD);N or Count(%)** | **SGLT2I users (N=21713) Mean(SD);N or Count(%)** | **DPP4I users (N=21713) Mean(SD);N or Count(%)** |  |
| ***Medications*** |  |  |  |  |  |  |  |  |
| SGLT2I frequency | 7.2(9.8);n=21713 | 7.2(9.8);n=21713 | - | - | 7.2(9.8);n=21713 | 7.2(9.8);n=21713 | - | - |
| DPP4I frequency | 5.2(7.4);n=39510 | - | 5.2(7.4);n=39510 | - | 6.6(6.7);n=21713 | - | 6.6(6.7);n=21713 | - |
| SGLT2I duration, days | 529.1(671.7);n=21713 | 529.1(671.7);n=21713 | - | - | 529.1(671.7);n=21713 | 529.1(671.7);n=21713 | - | - |
| DPP4I duration, days | 276.0(333.0);n=39510 | - | 276.0(333.0);n=39510 | - | 398.7(345.3);n=21713 | - | 398.7(345.3);n=21713 | - |
| Metformin | 54389(88.83%) | 20165(92.87%) | 34224(86.62%) | 0.21* | 40372(92.96%) | 20165(92.87%) | 20207(93.06%) | 0.01 |
| Sulphonylurea | 46909(76.61%) | 15218(70.08%) | 31691(80.21%) | 0.24* | 30961(71.29%) | 15218(70.08%) | 15743(72.50%) | 0.05 |
| Insulin | 30965(50.57%) | 11169(51.43%) | 19796(50.10%) | 0.03 | 22412(51.60%) | 11169(51.43%) | 11243(51.78%) | 0.01 |
| Acarbose | 1576(2.57%) | 880(4.05%) | 696(1.76%) | 0.14 | 1652(3.80%) | 880(4.05%) | 772(3.55%) | 0.03 |
| Thiozolidinedone | 11616(18.97%) | 6057(27.89%) | 5559(14.06%) | 0.34* | 11058(25.46%) | 6057(27.89%) | 5001(23.03%) | 0.11 |
| Glucagon-like peptide-1 receptor agonists | 1744(2.84%) | 1569(7.22%) | 175(0.44%) | 0.36* | 2632(6.06%) | 1569(7.22%) | 1063(4.89%) | 0.1 |
| Anticoagulants | 58001(94.73%) | 21711(99.99%) | 36290(91.85%) | 0.42* | 43422(99.99%) | 21711(99.99%) | 21711(99.99%) | 0 |
| Statins and fibrates | 29109(47.54%) | 15907(73.26%) | 13202(33.41%) | 0.87* | 29079(66.96%) | 15907(73.26%) | 13172(60.66%) | 0.27* |

**Supplementary Table 3. Univariable Cox regression to predict atrial fibrillation and stroke/transient ischemic attack before and after 1:1 matching.**

* for p≤ 0.05, ** for p ≤ 0.01, *** for p ≤ 0.001; HR: hazard ratio; CI: confidence interval; SD: standard deviation; SCD: sudden cardiac death; VF: ventricular fibrillation; VT: ventricular tachycardia; SGLT2I: sodium glucose cotransporter-2 inhibitor; DPP4I: dipeptidyl peptidase-4 inhibitor; ACEI: angiotensin converting enzyme inhibitors; ARB: angiotensin-receptor blockers; CV: coefficient of variation.

| **Characteristics** | **Before matching** |  | **After matching** |  |
| --- | --- | --- | --- | --- |
|  | **Atrial fibrillation**  **HR [95% CI];P value** | **Stroke/transient ischemic attack**  **HR [95% CI];P value** | **Atrial fibrillation**  **HR [95% CI];P value** | **Stroke/transient ischemic attack**  **HR [95% CI];P value** |
| ***Demographics*** |  |  |  |  |
| Male gender | 0.93[0.86-1.01];0.0686 | 1.04[0.96-1.13];0.3552 | 1.08[0.95-1.22];0.2423 | 1.34[1.19-1.51];<0.0001*** |
| Female gender | 1.0[Reference] | 1.0[Reference] | 1.0[Reference] | 1.0[Reference] |
| Baseline age, years | 1.08[1.08-1.09];<0.0001*** | 1.05[1.04-1.05];<0.0001*** | 1.08[1.08-1.09];<0.0001*** | 1.03[1.03-1.04];<0.0001*** |
| <50 | 1.0[Reference] | 1.0[Reference] | 1.0[Reference] | 1.0[Reference] |
| [50-60] | 0.26[0.23-0.30];<0.0001*** | 0.52[0.47-0.59];<0.0001*** | 0.42[0.36-0.49];<0.0001*** | 0.99[0.88-1.12];0.9206 |
| [60-70] | 0.67[0.61-0.74];<0.0001*** | 0.89[0.81-0.98];0.0202* | 1.29[1.14-1.47];0.0001*** | 1.11[0.98-1.26];0.0968 |
| [70-80] | 2.25[2.07-2.45];<0.0001*** | 1.76[1.60-1.93];<0.0001*** | 3.41[2.98-3.90];<0.0001*** | 1.67[1.43-1.94];<0.0001*** |
| >80 | 4.83[4.45-5.25];<0.0001*** | 2.75[2.49-3.04];<0.0001*** | 5.01[4.12-6.09];<0.0001*** | 2.49[1.95-3.17];<0.0001*** |
| ***Past comorbidities*** |  |  |  |  |
| Charlson’s standard comorbidity index | 1.46[1.43-1.48];<0.0001*** | 1.35[1.32-1.37];<0.0001*** | 1.52[1.47-1.56];<0.0001*** | 1.33[1.28-1.38];<0.0001*** |
| Diabetes with chronic complication | 2.14[1.64-2.78];<0.0001*** | 2.05[1.54-2.73];<0.0001*** | 2.61[1.81-3.78];<0.0001*** | 1.97[1.33-2.93];0.0008*** |
| Diabetes without chronic complication | 0.93[0.68-1.27];0.6417 | 1.40[1.07-1.84];0.0153* | 1.05[0.69-1.59];0.8374 | 1.46[1.04-2.05];0.0303* |
| Gastrointestinal bleeding | 1.33[1.06-1.68];0.0140* | 1.58[1.26-1.99];0.0001*** | 0.87[0.53-1.43];0.5888 | 1.53[1.07-2.19];0.0193* |
| Gout | 2.36[2.00-2.79];<0.0001*** | 2.08[1.72-2.51];<0.0001*** | 1.80[1.30-2.49];0.0004*** | 1.63[1.18-2.24];0.0027** |
| Heart failure | 4.53[3.95-5.19];<0.0001*** | 1.97[1.61-2.42];<0.0001*** | 5.47[4.39-6.81];<0.0001*** | 1.93[1.39-2.66];0.0001*** |
| Hyperlipidaemia | 0.86[0.66-1.11];0.2516 | 1.24[0.98-1.57];0.0688 | 0.75[0.51-1.10];0.1419 | 1.03[0.75-1.41];0.8496 |
| Hypertension | 1.77[1.63-1.92];<0.0001*** | 1.85[1.70-2.02];<0.0001*** | 1.82[1.60-2.07];<0.0001*** | 1.64[1.45-1.85];<0.0001*** |
| Hypoglycemia | 2.29[1.69-3.11];<0.0001*** | 1.34[0.88-2.04];0.1734 | 2.93[1.39-6.17];0.0046** | 2.22[1.00-4.96];0.0509 |
| Ischemic heart disease | 1.74[1.56-1.94];<0.0001*** | 1.35[1.19-1.54];<0.0001*** | 1.96[1.68-2.28];<0.0001*** | 1.31[1.12-1.55];0.0010** |
| Liver diseases | 0.62[0.44-0.87];0.0054** | 0.86[0.63-1.18];0.3522 | 0.73[0.48-1.12];0.1493 | 0.74[0.50-1.10];0.1371 |
| Acute myocardial infarction | 1.89[1.56-2.27];<0.0001*** | 1.38[1.09-1.73];0.0065** | 2.39[1.89-3.03];<0.0001*** | 1.95[1.53-2.48];<0.0001*** |
| Peripheral vascular disease | 1.69[1.17-2.44];0.0050** | 2.07[1.45-2.94];0.0001*** | 1.32[0.63-2.77];0.4678 | 3.22[2.04-5.06];<0.0001*** |
| Renal diseases | 2.44[2.00-2.97];<0.0001*** | 1.60[1.24-2.07];0.0003*** | 1.86[1.00-3.46];0.0518 | 0.97[0.43-2.16];0.9396 |
| VT/VF/SCD | 1.38[0.65-2.89];0.4001 | 1.12[0.47-2.69];0.8001 | 0.34[0.05-2.39];0.2756 | 0.90[0.29-2.79];0.8507 |
| Anaemia | 1.89[1.62-2.20];<0.0001*** | 1.65[1.38-1.96];<0.0001*** | 1.57[1.12-2.19];0.0091** | 1.64[1.20-2.24];0.0019** |
| Overweight | 0.62[0.34-1.11];0.1092 | 0.19[0.06-0.59];0.0042** | 0.64[0.34-1.19];0.1541 | 0.17[0.05-0.52];0.0020** |
| Cancer | 1.56[1.28-1.90];<0.0001*** | 1.45[1.16-1.81];0.0010** | 1.41[0.97-2.06];0.0706 | 1.48[1.05-2.09];0.0271* |
| Stroke/transient ischemic attack | 1.60[1.33-1.94];<0.0001*** | 5.30[4.68-6.01];<0.0001*** | 1.33[0.93-1.90];0.1181 | 6.01[5.03-7.19];<0.0001*** |
| ***Medications*** |  |  |  |  |
| SGLT2I v.s. DPP4I | 0.36[0.32-0.39];<0.0001*** | 0.38[0.34-0.43];<0.0001*** | 0.69[0.61-0.78];<0.0001*** | 0.50[0.44-0.57];<0.0001*** |
| SGLT2I frequency | 1.01[1.01-1.02];<0.0001*** | 1.01[1.01-1.02];<0.0001*** | 1.01[1.01-1.02];<0.0001*** | 1.01[1.01-1.02];<0.0001*** |
| DPP4I frequency | 1.00[1.00-1.01];0.2082 | 1.01[1.00-1.01];<0.0001*** | 1.02[1.01-1.03];<0.0001*** | 1.02[1.02-1.03];<0.0001*** |
| SGLT2I duration, days | 1.000[1.000-1.000];0.0003*** | 1.000[1.000-1.000];0.4706 | 1.000[1.000-1.000];0.0003*** | 1.000[1.000-1.000];0.4706 |
| DPP4I duration, days | 0.998[0.998-0.999];<0.0001*** | 0.999[0.999-0.999];<0.0001*** | 0.999[0.999-0.999];<0.0001*** | 0.999[0.999-0.999];<0.0001*** |
| Metformin | 0.48[0.44-0.53];<0.0001*** | 0.80[0.71-0.91];0.0005*** | 0.62[0.51-0.75];<0.0001*** | 0.84[0.68-1.04];0.1178 |
| Sulphonylurea | 1.12[1.02-1.23];0.0226* | 1.36[1.22-1.52];<0.0001*** | 1.11[0.97-1.27];0.1443 | 1.38[1.20-1.58];<0.0001*** |
| Insulin | 2.57[2.36-2.81];<0.0001*** | 2.69[2.45-2.96];<0.0001*** | 2.22[1.94-2.54];<0.0001*** | 2.43[2.14-2.77];<0.0001*** |
| Acarbose | 1.00[0.78-1.28];0.9713 | 0.93[0.70-1.22];0.5824 | 1.52[1.17-1.98];0.0019** | 0.86[0.62-1.19];0.3606 |
| Thiozolidinedone | 0.42[0.37-0.49];<0.0001*** | 0.66[0.58-0.74];<0.0001*** | 0.48[0.41-0.57];<0.0001*** | 0.92[0.81-1.05];0.2357 |
| Glucagon-like peptide-1 receptor agonists | 0.43[0.30-0.60];<0.0001*** | 0.43[0.30-0.62];<0.0001*** | 0.78[0.59-1.04];0.0878 | 1.09[0.87-1.38];0.4455 |
| Anticoagulants | 0.97[0.81-1.15];0.7137 | 1.44[1.16-1.80];0.0012** | - | - |
| Statins and fibrates | 0.44[0.40-0.47];<0.0001*** | 0.52[0.48-0.57];<0.0001*** | 0.79[0.70-0.89];0.0002*** | 0.79[0.70-0.89];0.0001*** |

**Supplementary Table 4. Univariable Cox regression to predict cardiovascular mortality and all-cause mortality before and after 1:1 matching.**

* for p≤ 0.05, ** for p ≤ 0.01, *** for p ≤ 0.001; HR: hazard ratio; CI: confidence interval; SD: standard deviation; SCD: sudden cardiac death; VF: ventricular fibrillation; VT: ventricular tachycardia; SGLT2I: sodium glucose cotransporter-2 inhibitor; DPP4I: dipeptidyl peptidase-4 inhibitor; ACEI: angiotensin converting enzyme inhibitors; ARB: angiotensin-receptor blockers; CV: coefficient of variation.

| **Characteristics** | **Before matching** |  | **After matching** |  |
| --- | --- | --- | --- | --- |
|  | **All cause mortality HR [95% CI];P value** | **Cardiovascular mortality HR [95% CI];P value** | **All cause mortality HR [95% CI];P value** | **Cardiovascular mortality HR [95% CI];P value** |
| ***Demographics*** |  |  |  |  |
| Male gender | 0.98[0.93-1.02];0.3275 | 1.13[1.03-1.23];0.0099** | 1.15[1.05-1.25];0.0017** | 1.25[1.03-1.52];0.0258* |
| Female gender | 1.0[Reference] | 1.0[Reference] | 1.0[Reference] | 1.0[Reference] |
| Baseline age, years | 1.09[1.09-1.10];<0.0001*** | 1.13[1.12-1.13];<0.0001*** | 1.08[1.08-1.09];<0.0001*** | 1.13[1.12-1.14];<0.0001*** |
| <50 | 1.0[Reference] | 1.0[Reference] | 1.0[Reference] | 1.0[Reference] |
| [50-60] | 0.26[0.24-0.28];<0.0001*** | 0.13[0.11-0.16];<0.0001*** | 0.44[0.40-0.49];<0.0001*** | 0.19[0.14-0.26];<0.0001*** |
| [60-70] | 0.53[0.49-0.56];<0.0001*** | 0.34[0.30-0.39];<0.0001*** | 1.10[1.00-1.20];0.0460* | 1.20[0.98-1.46];0.0747 |
| [70-80] | 1.97[1.87-2.08];<0.0001*** | 1.79[1.62-1.98];<0.0001*** | 3.07[2.79-3.37];<0.0001*** | 2.99[2.42-3.69];<0.0001*** |
| >80 | 6.67[6.35-7.00];<0.0001*** | 11.40[10.43-12.46];<0.0001*** | 6.96[6.17-7.83];<0.0001*** | 15.35[12.45-18.92];<0.0001*** |
| ***Past comorbidities*** |  |  |  |  |
| Charlson’s standard comorbidity index | 1.54[1.53-1.56];<0.0001*** | 1.61[1.58-1.63];<0.0001*** | 1.58[1.55-1.61];<0.0001*** | 1.70[1.64-1.76];<0.0001*** |
| Diabetes with chronic complication | 2.49[2.14-2.89];<0.0001*** | 1.82[1.32-2.51];0.0003*** | 2.39[1.84-3.11];<0.0001*** | 1.85[0.96-3.59];0.0669 |
| Diabetes without chronic complication | 1.66[1.44-1.92];<0.0001*** | 1.78[1.38-2.30];<0.0001*** | 1.73[1.38-2.18];<0.0001*** | 1.46[0.84-2.53];0.1806 |
| Gastrointestinal bleeding | 1.95[1.73-2.19];<0.0001*** | 2.55[2.10-3.09];<0.0001*** | 1.56[1.21-2.02];0.0006*** | 2.90[1.89-4.45];<0.0001*** |
| Gout | 2.44[2.21-2.70];<0.0001*** | 3.05[2.58-3.61];<0.0001*** | 1.88[1.51-2.34];<0.0001*** | 2.93[1.97-4.36];<0.0001*** |
| Heart failure | 4.00[3.66-4.37];<0.0001*** | 3.64[3.07-4.31];<0.0001*** | 4.61[3.93-5.42];<0.0001*** | 4.54[3.17-6.51];<0.0001*** |
| Hyperlipidaemia | 0.97[0.83-1.13];0.6826 | 0.97[0.74-1.28];0.8311 | 0.87[0.68-1.11];0.2598 | 0.91[0.54-1.55];0.7352 |
| Hypertension | 2.03[1.93-2.13];<0.0001*** | 2.19[2.00-2.40];<0.0001*** | 1.76[1.61-1.92];<0.0001*** | 1.68[1.37-2.04];<0.0001*** |
| Hypoglycemia | 3.59[3.07-4.19];<0.0001*** | 4.49[3.48-5.80];<0.0001*** | 2.78[1.64-4.70];0.0001*** | 4.93[2.04-11.90];0.0004*** |
| Ischemic heart disease | 1.57[1.46-1.68];<0.0001*** | 1.40[1.22-1.60];<0.0001*** | 1.67[1.50-1.86];<0.0001*** | 1.66[1.30-2.12];<0.0001*** |
| Liver diseases | 1.05[0.90-1.24];0.5380 | 0.75[0.53-1.06];0.1015 | 1.02[0.79-1.30];0.9061 | 0.62[0.31-1.25];0.1801 |
| Acute myocardial infarction | 2.06[1.84-2.30];<0.0001*** | 1.76[1.41-2.19];<0.0001*** | 2.29[1.94-2.71];<0.0001*** | 2.73[1.94-3.84];<0.0001*** |
| Peripheral vascular disease | 3.69[3.15-4.33];<0.0001*** | 3.31[2.44-4.50];<0.0001*** | 5.48[4.22-7.11];<0.0001*** | 4.16[2.15-8.05];<0.0001*** |
| Renal diseases | 4.45[4.04-4.89];<0.0001*** | 4.32[3.62-5.17];<0.0001*** | 3.47[2.53-4.77];<0.0001*** | 4.42[2.36-8.28];<0.0001*** |
| VT/VF/SCD | 2.48[1.75-3.51];<0.0001*** | 1.82[0.87-3.83];0.1124 | 3.33[2.14-5.17];<0.0001*** | 7.50[3.88-14.51];<0.0001*** |
| Anaemia | 3.16[2.93-3.41];<0.0001*** | 3.58[3.12-4.09];<0.0001*** | 2.36[1.95-2.86];<0.0001*** | 2.69[1.80-4.02];<0.0001*** |
| Overweight | 0.39[0.25-0.62];<0.0001*** | 0.14[0.03-0.56];0.0054** | 0.93[0.65-1.32];0.6713 | 0.00[0.00-Inf];0.9829 |
| Cancer | 2.36[2.13-2.61];<0.0001*** | 2.46[2.05-2.97];<0.0001*** | 2.99[2.48-3.59];<0.0001*** | 2.95[1.95-4.44];<0.0001*** |
| Stroke/transient ischemic attack | 2.26[2.05-2.50];<0.0001*** | 2.88[2.44-3.39];<0.0001*** | 2.37[1.96-2.86];<0.0001*** | 4.16[2.99-5.78];<0.0001*** |
| ***Medications*** |  |  |  |  |
| SGLT2I v.s. DPP4I | 0.16[0.14-0.17];<0.0001*** | 0.09[0.08-0.11];<0.0001*** | 0.34[0.31-0.37];<0.0001*** | 0.29[0.24-0.37];<0.0001*** |
| SGLT2I frequency | 1.02[1.01-1.02];<0.0001*** | 1.02[1.01-1.03];<0.0001*** | 1.02[1.01-1.02];<0.0001*** | 1.02[1.01-1.03];<0.0001*** |
| DPP4I frequency | 0.99[0.99-1.00];0.0003*** | 1.00[0.99-1.00];0.2822 | 1.02[1.02-1.03];<0.0001*** | 1.03[1.02-1.04];<0.0001*** |
| SGLT2I duration, days | 1.000[1.000-1.000];0.0028** | 1.000[0.999-1.000];0.0800 | 1.000[1.000-1.000];0.0028** | 1.000[0.999-1.000];0.0800 |
| DPP4I duration, days | 0.997[0.997-0.997];<0.0001*** | 0.997[0.997-0.997];<0.0001*** | 0.998[0.998-0.998];<0.0001*** | 0.998[0.997-0.998];<0.0001*** |
| Metformin | 0.27[0.26-0.29];<0.0001*** | 0.26[0.24-0.29];<0.0001*** | 0.36[0.32-0.40];<0.0001*** | 0.29[0.23-0.37];<0.0001*** |
| Sulphonylurea | 1.03[0.97-1.09];0.2744 | 1.29[1.15-1.44];<0.0001*** | 0.90[0.82-0.99];0.0265* | 0.93[0.76-1.15];0.5164 |
| Insulin | 5.21[4.88-5.55];<0.0001*** | 8.04[7.00-9.23];<0.0001*** | 5.56[4.94-6.25];<0.0001*** | 10.64[7.60-14.89];<0.0001*** |
| Acarbose | 1.05[0.90-1.21];0.5481 | 0.95[0.71-1.26];0.7180 | 1.29[1.06-1.57];0.0099** | 1.45[0.96-2.20];0.0741 |
| Thiozolidinedone | 0.37[0.34-0.40];<0.0001*** | 0.26[0.21-0.31];<0.0001*** | 0.53[0.47-0.59];<0.0001*** | 0.29[0.21-0.39];<0.0001*** |
| Glucagon-like peptide-1 receptor agonists | 0.11[0.07-0.17];<0.0001*** | 0.05[0.02-0.15];<0.0001*** | 0.25[0.18-0.34];<0.0001*** | 0.07[0.02-0.28];0.0002*** |
| Anticoagulants | 1.27[1.13-1.43];0.0001*** | 1.80[1.39-2.33];<0.0001*** | - | - |
| Statins and fibrates | 0.30[0.28-0.32];<0.0001*** | 0.25[0.22-0.28];<0.0001*** | 0.58[0.53-0.63];<0.0001*** | 0.48[0.40-0.58];<0.0001*** |

**Supplementary Table 5. Sensitivity analysis 1: One-year lag time applied.**

* for p≤ 0.05, ** for p ≤ 0.01, *** for p ≤ 0.001; SGLT2I: Sodium-glucose cotransporter-2 inhibitors; DPP4I: Dipeptidyl peptidase-4 inhibitors; HR: hazard ratio; CI: confidence interval.

| **Adverse outcomes** | **SGLT2I v.s. DPP4I**  **HR [95% CI];P value** |
| --- | --- |
| All-cause mortality | 0.32[0.28-0.37];<0.0001*** |
| Cardiovascular mortality | 0.27[0.19-0.37];<0.0001*** |
| Atrial fibrillation | 0.86[0.74-1.00];0.0366* |
| Atroke/transient ischemic attack | 0.66[0.56-0.76];<0.0001*** |

**Supplementary Table 6. Sensitivity analysis 2: Dfferent approaches based on the propensity score in the matched cohort.**

* for p≤ 0.05, ** for p ≤ 0.01, *** for p ≤ 0.001; SGLT2I: Sodium-glucose cotransporter-2 inhibitors; DPP4I: Dipeptidyl peptidase-4 inhibitors; HR: hazard ratio; CI: confidence interval; PS: propensity score; IPTW: inverse probability of treatment weighting, SIPTW: stable inverse probability of treatment weighting.

| **Outcomes** | **PS stratification**  **HR [95% CI];P value** | **PS with IPTW**  **HR [95% CI];P value** | **PS with SIPTW**  **HR [95% CI];P value** |
| --- | --- | --- | --- |
| All-cause mortality | 0.36[0.33-0.41];<0.0001*** | 0.35[0.24-0.57];0.0195* | 0.37[0.31-0.43];0.0009*** |
| Cardiovascular mortality | 0.29[0.22-0.39];<0.0001*** | 0.31[0.23-0.95];0.0229* | 0.32[0.24-0.95];0.0156* |
| Atrial fibrillation | 0.81[0.70-0.93];0.0032** | 0.86[0.74-1.00];0.0366* | 0.73[0.65-0.95];0.0246* |
| Stroke/transient ischemic attack | 0.53[0.47-0.61];<0.0001*** | 0.66[0.56-0.76];<0.0001*** | 0.09[0.02-0.42];0.0023** |
